# Hydrate Philly: An Intervention to Increase Water Access and Appeal in Recreation Centers

**DOI:** 10.5888/pcd17.190277

**Published:** 2020-02-20

**Authors:** Hannah G. Lawman, Sara Grossman, Xavier Lofton, Gregory Tasian, Anisha I. Patel

**Affiliations:** 1 Division of Chronic Disease and Injury Prevention, Philadelphia Department of Public Health, Philadelphia, Pennsylvania; 2 Center for Pediatric Clinical Effectiveness, The Children's Hospital of Philadelphia, Philadelphia, Pennsylvania; 3 Department of Biostatistics, Epidemiology, and Informatics, Perelman School of Medicine at the University of Pennsylvania, Philadelphia, Pennsylvania; 4 Department of Surgery, Division of Urology, Perelman School of Medicine at the University of Pennsylvania, Philadelphia, Pennsylvania; 5 Department of Surgery, Division of Urology, The Children’s Hospital of Philadelphia, Philadelphia, Pennsylvania; 6 School of Medicine, Stanford University, Stanford, California; 7 Philip R. Lee Institute for Health Policy Studies, University of California San Francisco, San Francisco, California

## Abstract

**Introduction:**

Previous interventions to increase water access and consumption have focused on school settings, have shown mixed results on sugar-sweetened beverage (SSB) consumption, and have rarely addressed tap water safety. Our randomized controlled trial examined how improving access and appeal of water in recreation centers in low-income neighborhoods affected counts of SSBs carried by youth attending summer camp.

**Methods:**

Recreation centers (N = 28) matched on their characteristics were randomly assigned to control or intervention groups. Intervention centers received a new water fountain with a bottle filler (hydration station), water testing services, reusable water bottles, and water promotion and education training and materials. Primary outcomes were 1-year changes in center-level average daily gallons of water from fountains and hydration stations (flowmeter readings). Secondary outcomes were counts of SSBs observed, use of bottled water and reusable water bottles, staff SSB consumption, and hydration station maintenance.

**Results:**

Results showed increased water use (*b* = 8.6, 95% CI, 4.2–13.0) and reusable bottle counts (*b* = 10.2, 95% CI, 4.2–16.1) in intervention centers compared with control centers. No change occurred in youth carrying SSBs at camp, but center staff’s past 30-day SSB consumption frequency decreased (*b* = −34.8, 95% CI, −67.7 to −1.9). Intervention sites had marginally lower odds of maintenance problems (OR = 0.09; 95% CI, 0.004–0.76, *P* = .06) than control sites.

**Conclusion:**

Although providing hydration stations along with water testing, reusable water bottles, education, and promotion increased water consumption among youth at recreation centers, it had no effect on the number of SSBs observed during camp. Future strategies to increase water consumption should also address reducing SSB intake.

**Summary**

**What is already known about this topic?**

Interventions in schools to install bottle-filling water fountains, called hydration stations, have yielded substantial increases in students’ water consumption but mixed results for changing sugar-sweetened beverage intake.

**What is added by this report?**

Recreation centers in Philadelphia with summer camp programs were randomly assigned to have a hydration station installed with the goal of increasing water intake and decreasing sugar-sweetened beverage consumption. Centers’ water use levels at fountains doubled, but no changes were observed in counts of sugar-sweetened beverage youth brought to camp.

**What are the implications for public health practice?**

Hydration stations may be a cost-effective strategy to increase water consumption in community recreation centers, but additional targeted strategies are needed to reduce sugar-sweetened beverage consumption.

## Introduction

The cognitive (1,2), physiological (3,4), and emotional (5,6) benefits of hydration are numerous and include better reaction time, improved memory, reduced risk of kidney stones, and improved mood. Furthermore, using water to replace sugar sweetened beverages (SSBs) aids in weight loss among adults (7,8) and prevents weight gain and aids in weight loss among youth (9–11). Concerns about the appeal and safety of tap water emphasize the need for water consumption interventions to address such concerns, particularly in low-income areas and racial/ethnic minority communities (12–14).

Previous interventions to increase access to and consumption of water have predominantly focused on school settings (15–19) or school-based after-school programs (20). These interventions have consistently shown significant and meaningful increases in youth water consumption overall and in low-income and racial/ethnic minority communities (15–17,19,20). However, studies have not examined interventions to increase water consumption in community recreation centers, which serve a large number of youth, provide water access to the community overall, and offer ideal settings for addressing youth’s risk for summer weight gain (21,22). In addition, publicly available bottle-filling stations may have additional benefits, such as reduced plastic bottle waste.

Our study aimed to test the effectiveness of an intervention to increase water use in recreation centers by improving water access and appeal through both built environment and sociocultural strategies in urban, low-income, and racially/ethnically diverse communities. We hypothesized that intervention sites would see greater water use than control sites.

## Methods

### Study design

Our study was a group-randomized controlled trial implemented in 28 Philadelphia Parks and Recreation Department (PPR) recreation centers. PPR programs predominantly serve children aged 6 to 12, though people of all ages use the centers. PPR centers serve over 2 million meals and snacks annually, emphasizing the need for access to appealing water. The study was conducted from July 2017 through August 2018.

Eligibility criteria for centers were 1) location in a low-income neighborhood as defined by having 20% or more of the residents in the center’s zip code at or below 100% of the federal poverty level, 2) having both summer school and after-school programs, 3) willingness to comply with the City of Philadelphia Healthy Vending Standards and to encourage youth not to bring in SSBs or “black bags” (black plastic bags characteristic of purchases from corner stores or “bodegas”), 4) water lines that were accessible and appropriate for installing hydration stations, 5) agreement to randomization, and 6) a potential matched site (Figure). Centers were assessed for these criteria and matched in pairs on center characteristics in the following order of priority: 1) type of facility and programs offered (eg, indoor/outdoor, sports league participation, pool, outdoor fields), 2) size of facility and programs conducted outside of schooltime, and 3) demographics of the census tracts surrounding the centers, including percentage of residents that were minority (ie, nonwhite) and percentage of residents with incomes below 100% of the federal poverty level. By using a public coin toss, 1 center in each matched pair was randomized to receive the intervention and the other to serve as the control. Immediately following random assignment, PPR committed to provide all control sites with a hydration station upon study completion. The Philadelphia Department of Public Health institutional review board approved the study.

**Figure:**
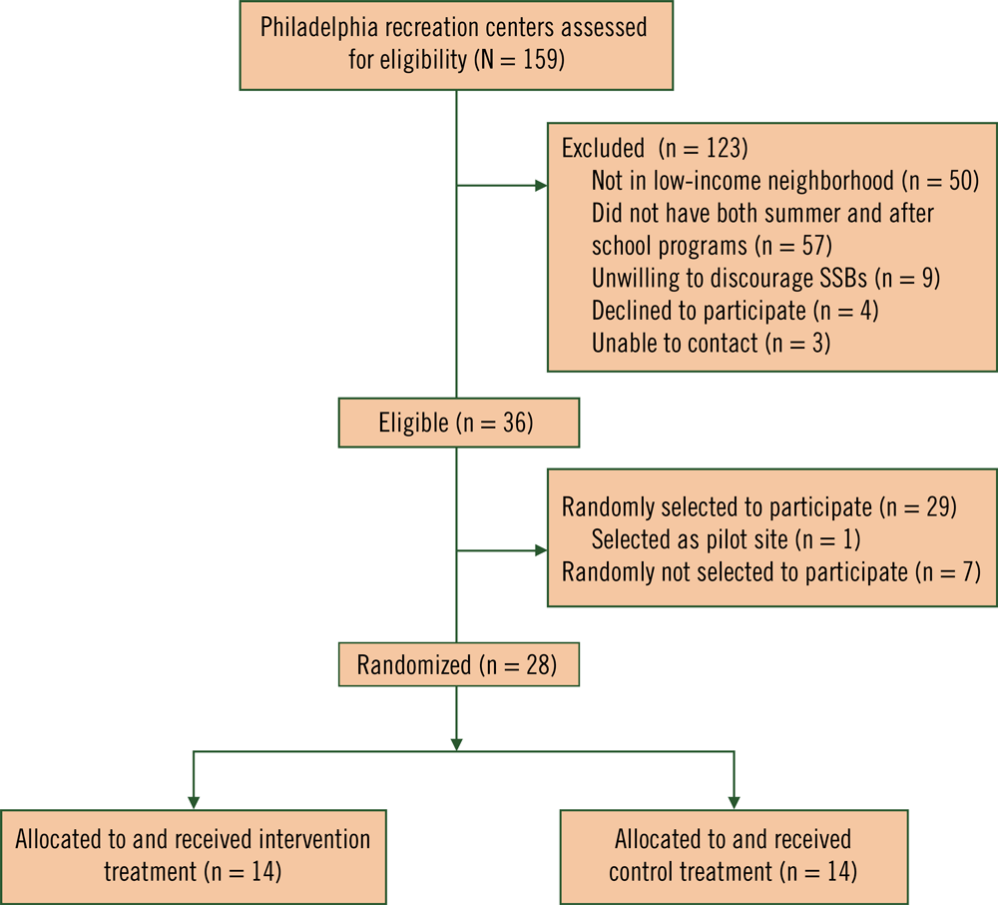
Hydrate Philly eligibility and enrollment flowchart.

A detailed description of our intervention and associated materials was published previously (23). Briefly, the Hydrate Philly intervention, developed with an emphasis on sustainability and scalability, was based on the socio-ecological model (24) and social cognitive theory (25); it emphasized improving environmental access to appealing water, reducing perceived barriers, improving efficacy, and changing social norms to improve beverage consumption patterns. The intervention’s objective was to increase the appeal of drinking water sources at recreation centers through the installation of new hydration stations (a water fountain with bottle filler) that were not rusty, broken, or dirty. Water quality concerns were addressed through water safety testing and communication of test results to the center’s staff, youth, and their families (Appendix). Because drinking water contaminants were not detected in center water systems, filters were not installed in hydration stations because of their cost and the need for their ongoing maintenance. Education and promotion consisted of distribution of reusable water bottles for youth and center staff members and modest programming and promotional efforts to encourage water and discourage SSB consumption (eg, a group-based “weekly water challenge” game, staff training [23]).

### Measures

The study included 2-week measurement periods, 1 at baseline (July–August 2017) and 1 post intervention (July–August 2018). Each weekday during each measurement period, 1 or 2 staff members per center, who were identified to serve as liaisons, self-reported their own beverage consumption and responded to daily 5-question, text-based surveys measuring water use; counts of SSBs, bottled water, and reusable bottles observed; and program attendance (23). The primary outcome was average daily gallons of fountain or station water used at each center as measured by objective flowmeter readings and reported to the research staff by text. At baseline, flowmeters (DigiFlow 6700M, 3/8” NPT) were installed in 1 water fountain per site. After the intervention, intervention sites reported volume readings from unit-installed flowmeters connected to the units’ bottle counters (ie, unit that converts fountain water used into number of 20-ounce bottles and shows measure on the unit’s display). These volume readings were converted to gallons. The first and last readings over each 2-week measurement period were subtracted and averaged over the total number of days to obtain center-level average daily gallons of water used.

As a secondary outcome, during measurement periods center staff members texted program attendance for the day and counted and reported the number of summer program participants observed with 1) an SSB, 2) single-use bottled water, and 3) a reusable water bottle. Daily reports were averaged to create 1 center-level estimate of average daily counts of camp youth with SSBs, bottled water, and reusable bottles and average daily program attendance.

Staff SSB consumption was measured before the start of each measurement period by using a previously validated beverage intake questionnaire (BEVQ-15) (26). This questionnaire was used to estimate the number of days in the past 30 days that an SSB or water was consumed (frequency) and the prevalence of daily SSB consumption.

From February through September 2018, recreation leaders and caretakers at all sites completed at least 3 surveys assessing time spent on cleaning and maintaining drinking water sources following station installations at intervention sites. Each center’s responses were averaged to create measures of time spent cleaning. Maintenance issues that arose in the previous week (eg, weak flow, nonoperational unit) that could not be resolved with on-site staff and required additional site visits by an off-site, specialized maintenance staff were described and used to determine whether sites reported any versus no maintenance issues. Maintenance surveys were collected only after hydration station installation (ie, no baseline data were collected).

To help corroborate staff-reported counts of beverages and water use as measured through flowmeters, research staff conducted water source observations post intervention for all center attendees (youth, staff, and visitors). The research staff used a standard protocol (23) based on existing measures (15,27) to observe fountain or station use and consumption of other beverages. Researchers conducted 30-minute observations of all sites at the same time on 5 separate days over the course of 1 to 2 weeks (140 observations). The research staff tallied fountain and station visits and timed how long they were in use; these measures were used to estimate ounces of water consumed, accounting for spillage (Appendix). The research staff also tallied counts of observed SSBs, bottles of water, and reusable bottles. For each measure, the average of the 5 daily observations was used in confirmatory analyses corresponding to text-based measures reported by center liaisons.

Fidelity was assessed in 2 ways: through self-reports by center liaisons and by independent observation. Post intervention, during the first week of summer camp, center leaders self-reported via a telephone call whether or not they had distributed water bottles, posted the weekly water challenge game for camp use, and distributed promotional flyers. The following week, research staff members visited sites to record the number of children seen with program-branded reusable water bottles and whether the game poster was displayed. Fidelity for fountain installation, water safety testing, and staff training was tracked by the research staff.

### Statistical analysis

Multiple linear (continuous outcomes) and logistic (binary outcomes) regression analyses were used to determine the effect of treatment assignment on each outcome, adjusting for covariates. The treatment effect *b* can be interpreted as the expected difference-in-differences between treatment and control sites post intervention. Secondary analyses for maintenance-related outcomes focused on differences between treatment and control groups post intervention only. Covariates were selected on the basis of a priori theoretical specification or whether they were significantly associated with the outcome. Covariates included baseline values of the outcome, the percentage of residents in the zip code that were nonwhite, average program attendance, and whether or not the site had a change in the number of operational water fountains over time (Appendix). A 2-sided *P* value of < .05 was the threshold for significance, and no adjustments were made for multiple comparisons.

## Results

Baseline site and participant characteristics were described previously (23). Sites had an average of 1.3 operational water fountains, 35 youth attending summer camp, and were in zip codes with approximately 34% of residents living at or below federal poverty level (Table 1). Participants were predominantly African American (64.3%) and aged 6 to 12 years (85.4%) (23). At baseline, staff members consumed SSBs 64.1 (standard deviation, 35.5) times per month, and 85.7% consumed SSBs at least daily. The most common SSBs were soda, 100% juice, and sweet tea (consumed 13.3–16.4 times per month). At baseline, no differences were observed across groups on participant or site characteristics, but the staff at intervention sites consumed significantly more SSBs and drank water significantly fewer times per month than the staff at control sites (*P* < .01 for both SSBs and water).

**Table 1:** Baseline Characteristics of Recreation Centers (N = 28) Participating in the Hydrate Philly Intervention, Philadelphia, Pennsylvania, July 2017–August 2018

Site Characteristics^a^	Treatment	Control	Total
No. of operational water fountains	1.2 (0.6)	1.4 (0.7)	1.3 (0.6)
Summer camp attendance	34.6 (19.3)	35.3 (13.2)	35.0 (16.2)
No. of full-time staff^ b^	1.5 (0.5)	1.4 (0.5)	1.5 (0.5)
No. of part-time or seasonal staff^ b^	2.6 (0.8)	2.7 (0.6)	2.6 (0.7)
Residents living at or below federal poverty level, % (SD)^c^	36.0 (14.4)	31.8 (10.9)	33.9 (12.7)
Racial/ethnic minority residents, % (SD)^c^	71.4 (19.9)	81.8 (16.3)	76.6 (18.7)

a Intervention and control sites did not differ significantly. Values are mean (standard deviation) unless otherwise indicated.

b Full-time staff members, usually center leaders, were typically older than part-time and seasonal staff members, who were usually young adults or high school students hired as temporary, part-time employees to support summer camp and after-school program activities.

c Determined using Census 2010 data for the zip code in which the recreation center is located.

**Primary outcome (recreation center water use).** From pre to post intervention at intervention sites compared with control sites, gallons of water used increased significantly when adjusted for covariates (*b* = 8.6; 95% CI, 4.2–13.0) (Table 2). Sensitivity analyses using observations of post-intervention ounces of water consumed also showed significantly higher water consumption in intervention sites than in control sites (*b* = 154.2; 95% CI, 32.9–275.6) (Table 3).

**Table 2:** Effect of the Hydrate Philly Intervention on Beverage Intake and Water Bottle Use in 28 Urban Recreation Centers, Philadelphia, Pennsylvania, July 2017–August 2018

Outcome	Unadjusted Means, Intervention Group, n = 14		Unadjusted Means, Control Group, n = 14		Adjusted Treatment Effect Estimate (95% CI)^a^	*P* Value^b^	Δ *r* ^2 c^
	Baseline	Post	Baseline	Post			
Center water source use (gallons/d)	7.9	14.6	10.6	9.7	8.6 (4.2 to 13.0)	<.01	.24
Youth SSBs^d^	7.1	7.6	7.0	6.5	0.2 (−6.5 to 7.0)	.95	0
Youth reusable bottles^d^	7.1	15.9	6.7	4.4	10.2 (4.2 to 16.1)	<.01	.15
Youth bottled water^d^	9.8	8.5	11.0	5.8	1.1 (−3.3 to 5.5)	.61	0
Youth single-use bottles^d^^,^ e	16.9	16.2	18.0	12.3	1.1 (−8.5 to 10.6)	.82	0
Staff water consumption (past 30-day frequency)^f^	47.3	55.8	85.0	85.3	8.3 (−17.6 to 34.1)	.53	0
Staff SSB consumption, frequency past 30-days^f^	82.2	67.8	47.0	67.3	−34.8 (−67.7 to −1.9)	.04	.06
Staff SSB consumption, daily prevalence, OR (95% CI)	94.1	82.4	77.8	78.9	0.24 (0.01 to 4.09)	.34	.02

Abbreviations: CI, confidence interval; SSB, sugar-sweetened beverage.

a Adjusted models controlled for baseline values, center attendance, percentage of neighborhood residents of nonwhite race/ethnicity, and whether the number of operational fountains changed between baseline and post.

b Significant at *P* < .05.

c Change in *r* ^2^ shows the additional variability accounted for when treatment assignment was added to the model.

d Average number of youth observed with SSBs, bottled water, and reusable bottles was taken from average daily counts reported by center liaisons during baseline and post measurement periods.

e Single-use water bottles were aggregated from counts of SSBs and bottled water.

f Indicates significant differences between intervention and control at baseline.

**Table 3:** Results From Sensitivity Analyses Using Post-Intervention Summer Camp Beverage Observations, Hydrate Philly Intervention, Philadelphia, Pennsylvania, July 2017–August 2018

Variable	Intervention, Unadjusted Mean (SD)	Control, Unadjusted Mean (SD)	Adjusted Group Comparison, *b* (95% CI)^a^
Observed water consumed, oz^b^	258.87 (170.70)	136.50 (119.92)	154.22 (32.85 to 275.6)^c^
Trips with water consumed, %^d^	0.26 (0.16)	0.28 (0.20)	0.02 (−0.13 to 0.17)
Observed count, youth sugar-sweetened beverages	1.11 (1.32)	2.11 (2.90)	−1.22 (−3.09 to 0.64)
Observed count, reusable bottles	1.16 (1.70)	0.81 (0.80)	−0.05 (−1.06 to 0.96)
Observed count, bottled water	3.20 (3.35)	2.36 (2.20)	0.11 (−2.13 to 2.34)
Observed count, single-use bottles	2.77 (2.17)	3.33 (3.66)	−1.16 (-3.63 to 1.31)

Abbreviations: CI, confidence interval; SD, standard deviation.

a Parameter estimates can be interpreted as the difference between treatment and control groups at post adjusting for covariates. Covariates were center water use at baseline (for average daily water consumed only), program attendance at post, percentage of neighborhood residents of nonwhite race/ethnicity, and number of operational indoor fountains at post.

b Observed estimates were calculated by averaging the 5 separate 30-minute observation periods for each outcome.

c Significant at *P* < .05.

d Calculated as the percentage of total trips past the water source that resulted in a person stopping to use the fountain.

**Secondary outcomes (youth intake of other beverages, staff beverage intake, intervention cost).** From pre to post intervention, a greater number of youth used reusable water bottles (*b* = 10.2; 95% CI, 4.2–16.1) (Table 2) at intervention sites than at controls. No significant intervention effects were found for youth bringing other beverages to centers, including SSBs, bottled water, or single-use bottles of any kind. By using averages from liaison-texted data, 7 youths (range 6.48–7.64) who attended (Table 2) brought SSBs, or approximately 20% of youth brought SSBs to summer camp. Sensitivity analyses, using post-intervention research staff observations, showed similar results with the exception that reusable water bottle use was no longer significant (Table 3). Staff at intervention sites reported consuming significantly fewer SSBs in the past 30 days (*b* = −34.8, 95% CI, −67.7 to −1.9) (Table 2). Staff water consumption patterns also improved, and although the odds of daily SSB consumption decreased in intervention centers following the intervention as compared with control sites, these results were not significant (Table 2).

Fountain and program costs (Table 4) show that maintenance cost savings in years 2 to 5 offset initial capital expenses to install new hydration stations. The odds of intervention sites reporting a maintenance problem were 91% lower compared with control sites (OR = .09; 95% CI, 0.004–0.76), but this was marginal (*P* = .06). Time spent on routine fountain cleaning was not significantly different across treatment and control sites post intervention (Table 5).

**Table 4:** Cost Estimates for an Average Treatment and Control Site Participating in the Hydrate Philly Intervention, Philadelphia, Pennsylvania, July 2017–August 2018^a^

Cost Category	6-Week Summer Program		Annual	
	Treatment	Control	Treatment	Control
**Drinking water source**				
Hydration station unit	872	0	872	0
Unit installation (labor)^b^	743	0	743	0
Water quality test^c^	128	0	128	0
Water source electricity^d^^,^ e	4	4	43	45
Water^d^^,^ f	3	2	26	17
Annual fountain maintenance (labor)^g^	5	46	44	395
Average total fountain cost year 1	1,755	52	1,855	457
Total estimated fountain cost years 2–5	47	207	449	1,827
Total cost years 1–5	1,802	258	2,304	2,283
**Program materials**				
Reusable water bottles	424	—	848	—
Promotional materials^h^	2,465	—	2,465	—
Staff training	614	—	614	—
Step stools and fountain floor mats	300	—	300	—
Total program materials year 1	3,803	—	4,227	—
Program materials years 2–5	1,697	—	3,393	—
Total cost years 1–5	5,500	—	7,620	—

Abbreviation: —, not applicable.

a All costs are reported in US dollars.

b Estimated actual average cost per center to replace existing fountains with hydration stations.

c Actual cost per center to test water quality, including lead in water.

d Summer program costs calculated for 30 program days based on use as observed in the study. Annual costs assumed use at same rate for 302 operational days.

e Based on intervention units using 370 W from Elkay Food Service specification sheet (available from authors on request), control units using an average of 390 W from specification sheets for existing units at baseline, and average price per kWh charged to study centers by their local electric utility. Daily hours of use at full capacity (1.1 h) was based on study data from observations, and remaining hours per day were assumed use at 10% rated watts.

f Based on average price per gallon charged to study centers by their local water supplier.

g Maintenance does not include filters and filters were not used in the Hydrate Philly study. Labor costs for maintenance were estimated from data in Table 5 and administrative records of plumbing staff site visits for maintenance issues that could not be resolved with on-site building maintenance staff.

h Includes cost of graphic design services, group-game posters, and parent and center handouts.

**Table 5:** Differences in Drinking Water Source Cleaning and Maintenance Issues in Hydrate Philly Intervention and Control Sites, Hydrate Philly Intervention, Philadelphia, Pennsylvania, July 2017–August 2018

Water Fountain Maintenance	Intervention	Control	Adjusted treatment effect (95% CI)^a^	*P* Value
Routine cleaning, mean (SD)	2.25 (0.54)	2.45 (0.53)	−0.12 (−0.55 to 0.30)	.56
Extensive cleaning, mean (SD)	1.13 (0.13)	1.24 (0.20)	−0.08 (−0.22 to 0.05)	.23
Time spent cleaning, min per week, mean (SD)	3.03 (2.58)	3.58 (3.25)	−0.37 (−2.82 to 2.08)	.76
Sites with a maintenance issue, n (%)^b^	1 (14.3)	7 (50.0)	0.09 (0.004 to 0.76)	.06

a Parameter estimates (or odds ratio for sites with a maintenance issue) can be interpreted as the difference between treatment and control groups at post, adjusting for covariates. Adjusted models controlled for number of operational indoor fountains at post and level of on-site maintenance employee support (full-time, part-time, none).

b Sites reporting maintenance issues required site visits from separate centralized trades union plumbing staff members because of issues unable to be resolved by on-site maintenance employees.

**Intervention fidelity.** Results from the 14 intervention sites showed high compliance (71.4%–100%) with most intervention components except for use of the group-based water promotion game (57.1%) (Table 6). The key environmental changes that represent the most sustainable and scalable components (station installation, water quality testing, reusable water bottle distribution) showed perfect fidelity, whereas the program components (educational materials, water promotion game, staff training) showed moderate fidelity (50.0%–92.9%). Most sites (64%) reported that children took the water bottles home though some requested bottles be left on site (7%) until the end of summer camp or allowed children to do either (29%).

**Table 6:** Results of Intervention Fidelity Assessment at the 14 Intervention Sites, Hydrate Philly Intervention, Philadelphia, Pennsylvania, July 2017–August 2018

Fidelity Measure	Site Characteristic	No. Centers Meeting Fidelity^a^
Hydration station installed	Received at least 1 new water fountain with bottle-filling station	14
Passed water quality testing	Tested water for quality and safety and found all results within a safe range	14
Attended training	Site leaders attended the training on water promotion and sugar-sweetened beverage control strategies. Those who did not attend received a one-on-one training at a later date.	10
Distributed branded reusable water bottles	Reported distributing the branded water bottles to summer camp participants	14
Branded reusable water bottles observed in use	Had at least 1 branded bottle observed during any of the 5 observation days	11
Used group-based promotional game	Observed to display the promotional game in their center or reported using it prior to observations	7
Distributed educational materials	Reported distributing the educational materials to parents and/or community members	13
Fidelity score, mean (standard deviation)	One point awarded for meeting each of the above criteria. Scores range from 0 to 7.	5.93 (0.92)

a Values are numbers unless otherwise indicated.

## Discussion

Results of our randomized controlled trial to test the effect of Hydrate Philly led to several key study findings. First, average daily gallons of water used nearly doubled and water consumption was more than twice as high in intervention sites than in control sites. These findings are consistent with previous studies that evaluated the effect on water consumption of installation of safe and appealing water sources in schools and after-school programs (15,17,19,20). Interventions to improve water access in summer programs in parks and recreation centers complement such programs in schools for promoting water consumption and its numerous physical, psychological, and cognitive outcomes. Such interventions are particularly important in low-income, racial/ethnic minority communities, whose residents have been shown to drink less tap water (12–14) and to be less hydrated (28) than their higher-income and nonminority peers. The large increase in center water use is encouraging given the concerns about water quality expressed by many recreation center staff members (23). This could be related to the improved appeal of the stations, water quality testing, promotion of results, or all.

Another key finding was that SSB consumption by youth did not change significantly following the intervention. An estimated 20% of youth each day brought an SSB to centers. Previous school-based studies are mixed in finding an effect of increased water access on SSB consumption with some finding no change (16,17,19) and others finding a decrease (15,18,20). Two studies showed a benefit to youths’ weight status following water source interventions: 1 study suggested reduced energy intake from beverages (18) whereas another did not (19).

The limited effect on SSB intake among youth observed in our study could be related to implementation of the Philadelphia beverage tax 7 months before the study began, which led to large, immediate decreases in SSB sales (29). Further reduction in youth SSB consumption in our study may have required a strong SSB-targeted intervention. We encountered challenges in obtaining buy-in from centers, parents, and youth to implement policies to ban black bags and competing beverages during summer camp. Although a ban on SSBs or outside competing foods (ie, prohibiting the sale of outside foods and beverages available for purchase at recreation centers) is appealing from a public health perspective, this approach was not feasible in our study and received pushback when previously attempted at PPR.

Our study did, however, find patterns of reduced SSB consumption and increased water consumption among staff members, which was not measured in other water source interventions. Staff consumption of healthy beverages may be beneficial for role modeling, social norms, and environmental context of youths’ beverage consumption (eg, peer/staff influences, expectations for camp (24,25). For example, students whose teachers drank water in front of their class have been shown to be more likely to drink water during the day (30).

Intervention sites had 91% lower odds of having maintenance problems with fountains and stations, did not report increased time spent cleaning water sources, and were projected to save enough on maintenance costs after approximately 5 years to offset the initial capital cost of new hydration stations. This was likely due to the very old age of the existing water fountains, which required extensive maintenance. Although only marginally significant, such reductions in maintenance costs are of practical importance in a setting with limited resources. Only one intervention site experienced a maintenance problem during the study, whereas half of the control sites reported recurring leaks, clogs, or other problems requiring fountains to be shut off and the plumbing staff to make site visits. Previous studies have not evaluated maintenance changes after water source interventions, but a related study demonstrated their cost-effectiveness in school settings (17). Concern about maintenance costs for new equipment may be a barrier when considering water infrastructure improvements. However, results suggest sufficient maintenance cost savings from replacing aging infrastructure with new water stations to offset installation costs, which could generate organizational support for replacement.

Lastly, the increase in use of reusable bottles at intervention sites suggests that they were an effective tool for increasing water consumption. However, overall use of reusable water bottles remained relatively low, and sensitivity analyses of use of reusable bottles was similar in intervention and control sites. This indicates that children did not always bring their reusable bottles to camp and that use was intermittent. Consistent with previous studies that made water containers available on site (15,17,19,20), future studies might consider having youth leave the reusable water bottles at centers to reduce leaving them at home. We saw no changes in use of bottled water or all single-use bottles, which could have been reused at the bottle filler in intervention sites. No previous studies have examined the effect of water source interventions on plastic bottle waste; however, this is an outcome of interest for organizations that are increasingly focusing on sustainability goals. The increased use of reusable bottles in the current study is encouraging, though future research might consider more sensitive measures (eg, assessing the number of plastic bottles entering the waste stream).

This study was novel in its examination of the effect of a scalable, sustainable intervention to promote water access and consumption in nonschool settings and by its inclusion of outcomes relevant to agencies and policy makers considering infrastructure changes, such as staff beverage consumption, use of reusable bottles, and water fountain maintenance. However, our study had limitations. First, the study lacked individual-level outcomes on youth drinking behaviors and did not distinguish between youth and staff water use. However, center-level water use is believed to be a reasonable approximation. Per capita water estimates were not appropriate because the use of fountains by groups other than the youth at summer camp (eg, participants in other community and sports programs), which limits comparability of center water use and consumption estimates. Second, maintenance and observation data were only available post intervention. Third, staff beverage consumption was self-reported, which may have been subject to response bias. All other study data support baseline equivalency across treatment groups with the exception of staff beverage consumption. Fourth, different flowmeters were used in intervention and control sites post intervention for the primary outcome measure, and their comparability is unknown. It is unlikely that different commercial flowmeters produce measurements that are meaningfully different over time, and results were reinforced by post-intervention observations, which found similar effect sizes. Fifth, because of resource constraints, flowmeters were only installed on 1 fountain per center that was in close proximity to the youth summer camps. Because the number of fountains in intervention versus control sites was the same, the number of fountains per center was not likely to lead to systematically different use of drinking water sources (ie, differences are likely to be attributed to random error). Lastly, our study was based on the primary outcome of center water use, so positive changes in some secondary outcomes may have been underpowered.

Our intervention to promote water access and appeal in public recreation centers in low-income communities of racial/ethnic minorities significantly increased center water use and use of reusable water bottles, decreased staff SSB consumption, and significantly reduced water fountain maintenance. Because the intervention had no effect on youth SSB consumption, reducing youth SSB intake at recreation centers may require multiple targeted strategies. Study results can inform stakeholder and policy maker decisions about how to prioritize water access and water appeal infrastructure projects and inform larger studies to examine potential long-term health effects of such interventions.

## Appendix

**Water fountain appeal and water quality testing results.** All sites received baseline needs assessments, which documented the observed conditions of all existing water fountains in intervention and control sites. The primary fountain, which had the flow meter installed, was recorded as having satisfactory water flow in 27 of 28 sites. A total cleanliness score, composed of observations regarding water flow, debris, trash, hair, food, mold, gum, insects, bodily fluids, or other unsanitary items located on the fountain or obstructions to the fountain (eg, mop bucket), was calculated. Overall, 50% of sites received a perfect cleanliness score (7 intervention, 7 control). The most common cleanliness problem observed was rust (5 intervention and 4 control sites). Staff members reported barriers to youth drinking more water as no barriers (n = 12), lack of access/inconvenient location (n = 5), and children’s preferences for other drinks (n = 3). Comparable levels of the recreation center leader’s confidence in the safety of the water were reported across intervention and control sites (“somewhat or very confident” in water safety endorsed by 78.6% and 64.3% of intervention versus control sites, respectively) (23). Water safety testing was conducted only in intervention sites as part of the intervention primarily to address perception of water quality but also to ensure that the water that was promoted for consumption was safe (23). All water safety and quality measures for centers were within normal and safe ranges.

**Description of confirmatory and sensitivity analyses. **The research staff conducted 30-minute observations at each matched intervention and control site at the same time on 5 separate days (140 observations total). Research staff members stood near enough to the fountains/stations to have a line of sight to the spigots but not so close as to feel intrusive. Staff members could not distinguish summer camp youth from other center visitors and therefore tallied all individuals observed (youth, staff, visitors). Because observations occurred during summer camp programs, observations most likely reflect summer camp youth and a small number of center staff members. The research staff tallied any observed SSBs in the vicinity of the fountain/station as well as visits to the fountain/hydration station and used a stopwatch to time how many seconds (to 1/10 second) the water source was being used during each use. The volume of water consumed during the 30-minute observation period was calculated by using existing methods (15). Specifically, for each observation period the total time that water was being consumed was aggregated, multiplied by the flow rate of the fountain, and then multiplied by a waste factor established in a previous study (ie, 0.32 for spigots, 0.96 for bottles/cups) to account for spillage (eg, wasted water).

Although center-level water use as measured by flow meters and center level water consumption examined by research staff observations are related measures, they are not directly comparable. Center-level water use assessed by flowmeters captured all use of that specific fountain for an entire day, including use by other community and sports programs at the facility as well as wasted water. Per capita estimates using these data would be inappropriate because study measures captured only youth summer camp participation, not broad center-level attendance including other programs, and would thus over-inflate youth per capita water consumption estimates.

In contrast, center-level water consumption estimates examined through observations apply only to a typical 30-minute period during summer camp and removed estimated wasted water. Thus, these data may be appropriate for estimating per capita consumption during summer camp with the limitation that individual consumption was not tracked.
